# RAR and infant mortality: a crucial link in cardiac surgery outcomes

**DOI:** 10.3389/fimmu.2026.1806409

**Published:** 2026-04-16

**Authors:** Qi Yang, Qi Liu, Xing Zhong, Dandong Luo, Chongjian Zhang

**Affiliations:** Department of Cardiac Surgical Intensive Care Unit, Guangdong Provincial People’s Hospital (Guangdong Academy of Medical Sciences), Southern Medical University, Guangzhou, China

**Keywords:** cardiac surgery, infant, in-hospital mortality, red cell distribution width-to-albumin ratio (RAR), relationship

## Abstract

**Background:**

In-hospital mortality remains a significant concern in infant cardiac surgery, yet precise risk stratification tools are lacking. The prognostic value of the red cell distribution width-to-albumin ratio (RAR)—a composite marker of inflammation and nutritional status—remains unestablished in this population. This study aimed to examine the association between RAR and in-hospital mortality in infants undergoing cardiac surgery.

**Methods:**

This work constitutes a retrospective observational cohort analysis. The study population comprised infants admitted to Guangdong Provincial People’s Hospital for cardiac surgery from January 2017 through October 2021.The optimal cut-off value for the RAR was determined to be 1.35 fL/(g/L) using the Youden index method. Based on this threshold, all participants were categorized into a high-RAR group and a low-RAR group. The relationship between RAR levels and mortality during hospitalization was then examined through multiple analytical approaches, including logistic regression, subgroup analyses, and modeling with restricted cubic splines (RCS).The predictive performance of the RAR was further evaluated through an analysis of the receiver operating characteristic (ROC) curve.

**Results:**

Mortality rates in this surgical cohort increased with higher RAR values. A consistent relationship linking the RAR to mortality was observed regardless of whether this biomarker was incorporated into statistical models as a continuous measure or a categorical parameter. Among 3634 patients, logistic regression analysis indicated that infants with RAR > 1.35 fL/(g/L) had a significantly higher mortality risk compared to those with RAR ≤ 1.35 fL/(g/L) (OR = 2.16, 95% CI 1.01-4.60, p < 0.05). Furthermore, the direction of the association was consistent across subgroups. The predictive performance of the model was good, with an area under the ROC curve of 0.8.

**Conclusion:**

A higher RAR—specifically a value above 1.35 fL/(g/L)—was associated with increased mortality risk during hospitalization in infants undergoing cardiac surgery. As an easily measurable blood-based indicator, RAR demonstrates potential value for clinical practice and research applications.

## 1 Introduction

Congenital heart disease (CHD) is the most prevalent birth defect, accounting for approximately one-third of all major congenital anomalies (1). Despite advancements in surgical care that have reduced mortality over recent decades, CHD remains a leading cause of birth defect-related deaths and infant mortality (2).

Among global regions, Asia exhibits the highest birth frequency of CHD, with an estimated incidence of 9.3 per 1,000 live births, compared to 8.2 in Europe and 6.9 in North America (1, 3). Approximately 25% of children with CHD require surgery within their first year of life (4), and pediatric CHD surgeries are associated with significant risks, with approximately 4.2% resulting in in-hospital death (5). Within the 0–14-year age group, 80.5% of CHD-related deaths occur in children under 1 year old (6).

Elevated red cell distribution width(RDW) indicates significant disruptions in erythrocyte homeostasis and is closely linked to various diseases and their outcomes. This marker demonstrates a positive association with an increased likelihood of mortality from all causes and from cardiovascular-specific events (7). The elevation of RDW serves as an indicator of compromised erythropoiesis and alterations in erythrocyte longevity, conditions frequently correlating with a range of metabolic dysfunctions, which include telomere attrition, oxidative stress, inflammatory processes, nutritional deficiencies, red blood cell hemolysis, and other pathological alterations (8). Albumin(ALB) contributes to the modulation of inflammation, maintenance of vascular endothelial integrity, and preservation of acid–base balance; lower albumin levels are associated with poorer prognosis (9).

Previous studies have often analyzed RDW and ALB in combination, as their integration provides a comprehensive understanding of health conditions linked to mortality risk (10). Research specifically focused on RAR in infants remains limited. Therefore, investigating the potential association between this biomarker and mortality outcomes is justified in infants aged 0–365 days who underwent cardiac surgery. Indeed, the scarcity of research on RAR in this population makes it reasonable to explore its relationship with mortality.

## 2 Methods

### 2.1 Study participants and grouping

The study included 5,156 infants, who were defined as patients under one year of age and had undergone cardiac surgical procedures. These surgeries were performed at Guangdong Provincial People’s Hospital from January 2017 to October 2021.Following surgery, all infants were transferred to the specialized cardiac intensive care unit for postoperative monitoring and management.

**Figure 1:**
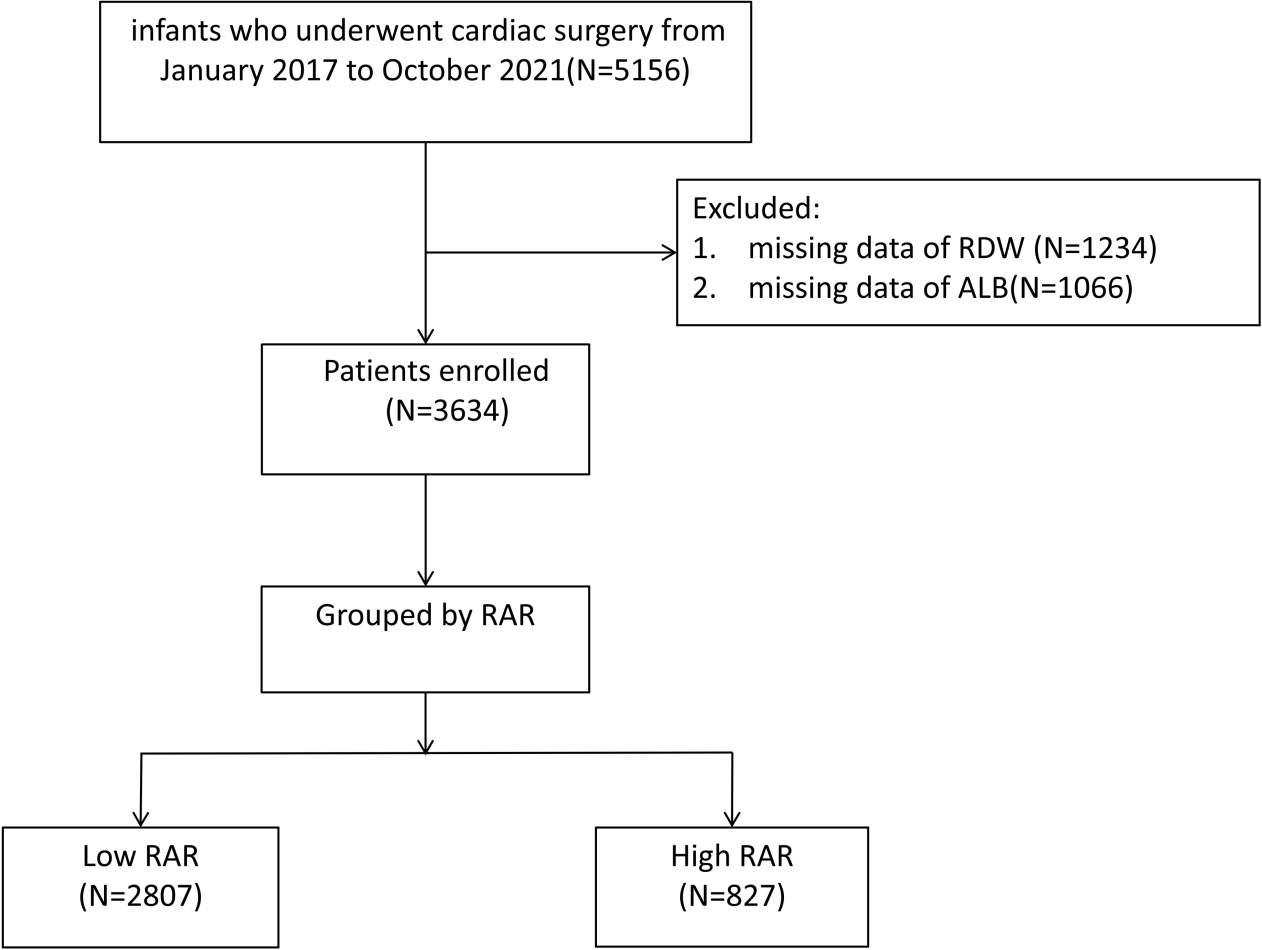
Study flow diagram

The initial diagnosis of CHD involves clinical practitioners evaluating each patient’s unique symptoms and physical signs. Definitive confirmation relies on non-invasive imaging techniques, particularly echocardiography and computed tomography (CT) scanning. Furthermore, in the context of patients with complex congenital heart disease, auxiliary 3D cardiac reconstruction imaging or pulmonary arteriography can be integrated into the preoperative evaluation procedure to enhance diagnostic accuracy. Cardiac magnetic resonance imaging (MRI) is additionally performed for those patients who are identified with severe impairment of myocardial contractile function. The study’s inclusion criteria encompassed two key aspects: (1) an age range of 0 to 365 days, and (2) a history of cardiac surgical intervention. For exclusion criteria, patients with missing data regarding RDW or ALB were excluded from the study. The final study cohort included 3,634 eligible patients, with in-hospital mortality as the study endpoint. Study data were extracted from medical records for a retrospective analysis, to ensure no risk to the children’s privacy. The research protocol (Approval Code: KY-Z-2022-311-04) received formal approval from the Medical Research Ethics Committee at Guangdong Provincial People’s Hospital. The study flow is illustrated in **Figure 1**.

### 2.2 Data collection

A retrospective review of medical treatment records was performed to collect baseline characteristics, including demographic and laboratory data. Demographics include age, gestational age, gender, height, weight. Laboratory parameters assessed were red blood cells (RBC), white blood cells (WBC), platelets (PLT), glucose (GLU), Creatinine (CREA), red cell distribution width standard deviation (RDW-SD), and albumin (ALB). Blood samples were collected upon admission, prior to any albumin infusion, blood transfusion, or fluid resuscitation during this hospitalization.

The recorded operative variables comprised three key parameters: the application of cardiopulmonary bypass (CPB), the total duration of CPB, and the time of aortic cross-clamping (AOX). Furthermore, documentation was completed for both the Society of Thoracic Surgeons-European Association for Cardio-Thoracic Surgery (STS-EACTS) mortality score and the confirmed diagnosis of CHD. The complexity of each surgical procedure was evaluated using the STAT Mortality score (11).

Compared to RDW coefficient of variation(RDW-CV), the RDW-SD is regarded as a more precise measure for quantifying variations in erythrocyte dimensions, as it captures a broader range of red cell volume distribution and thus better reflects erythrocyte volume heterogeneity (12).

RDW was represented by RDW-SD (fL), and albumin concentrations were recorded in g/L.RAR was derived using the formula:RAR (fL/(g/L))=RAR/ALB.

### 2.3 Statistical analysis

The present analysis examined differences in baseline profiles and relevant clinical parameters across patient groups stratified according to their RAR levels. For categorical variables, frequencies and percentages were calculated. Group differences were then assessed using the Chi-square or Fisher’s exact test. For continuous variables, the Kolmogorov-Smirnov test was first applied to assess their distribution pattern. Normally distributed data are presented as mean ± standard deviation (SD), and non-normally distributed data are presented as median with interquartile range (IQR).Intergroup comparisons were subsequently performed using either the independent samples t-test or the Mann–Whitney U test, depending on data characteristics. Missing values (**Supplementary Table 1**) were handled using random forest imputation, and all subsequent multiple model analyses were performed using the imputed dataset.

To account for potential confounding, we constructed a series of multivariable regression models using a hierarchical approach. Covariates for adjustment were selected based on their clinical relevance and established associations with the outcome as identified in prior literature. To ensure the stability of our models, we assessed both pairwise correlations and multicollinearity. Variables demonstrating an apparent correlation with another covariate or exhibiting a variance inflation factor (VIF) exceeding 5 were considered problematic and were excluded from the analysis (**Supplementary Table 2**). Initially, a univariable logistic regression was fitted to evaluate the unadjusted relationship between each variable and mortality. A multivariate logistic regression analysis was performed to evaluate the association between RAR and in-hospital mortality in infants, with outcomes expressed as odds ratios (OR) and 95% confidence intervals (CI). An optimal cut-off value for differentiating between low and high RAR was determined using a receiver-operating characteristic (ROC) curve and the Youden index method.

The analytical sequence commenced with Model 1,which did not incorporate any covariates. Subsequently, Model 2 was constructed with partial adjustment, including the variables of sex, age, gestational age, height, and weight. The most extensive adjustment was implemented in Model 3, which accounted for a broader set of factors: sex, age, gestational age, height, weight, STS-EACTS score, use of CPB, AOX time, diagnosis of cyanotic CHD, and the concentrations of WBC, RBC, platelets, and glucose. Subgroup analyses evaluated the consistency of the RAR-mortality relationship across clinically relevant subgroups, in parallel, the dose-response association linking RAR levels to the risk of in-hospital death was explored through RCS modeling.

We assessed the predictive accuracy of the RAR for in-hospital mortality using the area under the receiver operating characteristic curve (AUC). To determine whether RAR offered incremental predictive value, we used the DeLong test to compare the AUC of the STS-EACTS score alone with that of a combined model that included both the STS-EACTS score and RAR.

All statistical computations in this study were performed with R software(version 4.1.0),with statistical significance defined as p < 0.05.

## 3 Results

### 3.1 Patient characteristics

The study included 3,634 eligible participants, with an RAR cutoff value of 1.35fL/(g/L) determined by optimizing the ROC curve’s specificity and sensitivity. The cohort was stratified into low- and high-RAR groups. Compared with the low-RAR group, infants in the high-RAR group were younger (40.00 [16.00–76.00] vs. 170.00 [101.00–236.00] days, P < 0.001), and exhibited smaller height (50.00 [50.00–55.00] vs. 63.00 [57.00–68.00] cm, P < 0.001) and weight (3.50 [3.00–4.25] vs. 6.00 [4.85–7.00] kg, P < 0.001). Laboratory results showed lower levels of RBC, GLU, PLT, and ALB in the high-RAR group. Regarding surgical management, the high-RAR group had longer CPB and AOX durations. The in-hospital mortality rate was higher in the high-RAR group than in the low-RAR group (5.20% vs 0.68%,P<0.001) (**Table 1**).

**Table 1:** Baseline characteristics of participants with low and high RAR.

Characteristic	Low RAR(≤ 1.35)	High RAR (>1.35)	P-value
N	2807	827	
Age (day)	170.00(101.00-236.00)	40.00 (16.00-76.00)	<0.001
Male			0.371
No	1063 (37.87%)	299 (36. 15%)	
Yes	1744 (62. 13%)	528 (63.85%)	
Gestational age(week)	39.00 (38.00-40.00)	39.00 (37.00-40.00)	<0.001
Height (cm)	63.00 (57.00-68.00)	50.00 (50.00-55.00)	<0.001
Weight (kg)	6.00 (4.85-7.00)	3.50 (3.00-4.25)	<0.001
WBC (x10^9/L)	9.61 (7.83-11.73)	9.46 (7.72-11.94)	0.25
RBC (x10^12/L)	4.31 (3.84-4.74)	3.83 (3.37-4.30)	<0.001
GLU (mmol/L)	5.07 (4.48-5.73)	4.83 (4.13-5.75)	<0.001
PLT (x10^9/L)	368.00 (300.50-444.00)	334.00 (262.00-434.50)	<0.001
CREA (μmol/L)	21.50 (17.29-31.52)	33.24 (23.61-44.40)	<0.001
RDW-SD(fL)	39.60 (36.90-43.30)	54.30 (50.10-59.35)	<0.001
ALB(g/L)	40.60 (38.50-42.90)	33.59 (31.23-36.00)	<0.001
RAR(fL/(g/L))	0.97 (0.88-1.11)	1.59 (1.45-1.79)	<0.001
Cyanotic CHD diagnosis			<0.001
No	2121 (75.56%)	486 (58.77%)	
Yes	686 (24.44%)	341 (41.23%)	
STS-EACTS score	3.00 (2.00-3.00)	3.00 (2.00-4.00)	<0.001
CPB			<0.001
No	235 (8.37%)	137 (16.57%)	
Yes	2572 (91.63%)	690 (83.43%)	
CPB time(min)	74.00 (57.00-101.50)	87.00 (57.00-142.00)	<0.001
AOX time(min)	39.00 (28.00-57.00)	44.00 (25.00-76.00)	<0.001
In-hospital mortality			<0.001
No	2788 (99.32%)	784 (94.80%)	
Yes	19 (0.68%)	43 (5.20%)	

RBC, red blood cells; WBC, white blood cells; PLT, platelets; GLU, glucose; RDW-SD, red cell distribution width standard deviation; ALB, albumin; RAR, red cell distribution width-to-albumin ratio; CREA, Creatinine; STS-EACTS, Society of Thoracic Surgeons-European Association for Cardio-Thoracic Surgery; CPB, cardiopulmonary bypass; AOX, aortic cross-clamping.

### 3.2 Curve fitting with restricted cubic splines for smoothing

**Figure 2:**
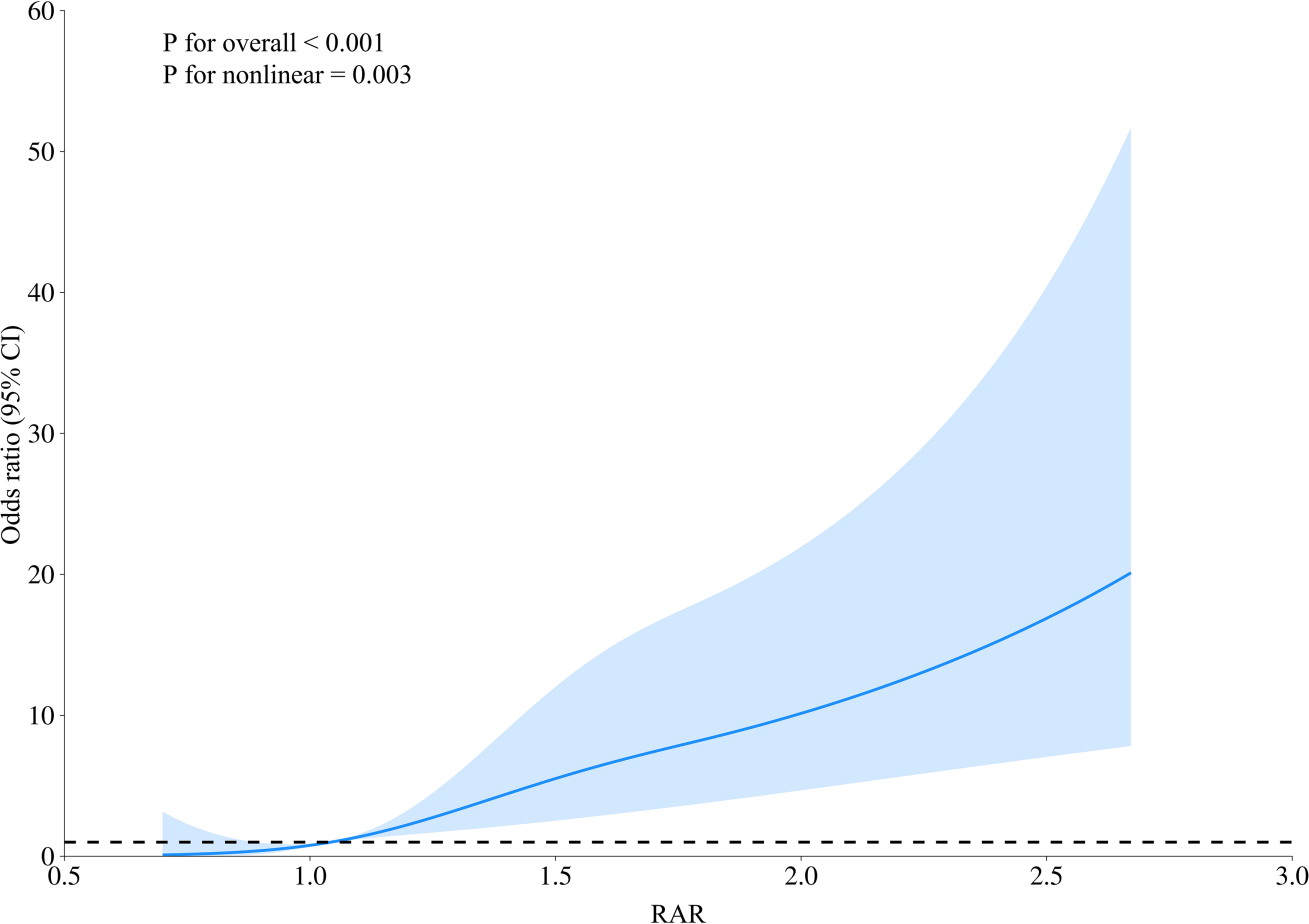
Analysis of the link between RAR and in-hospital mortality risk based on the RCS Model.

The fully adjusted RCS analysis demonstrated a significant nonlinear positive relationship between RAR and in-hospital mortality risk (p < 0.05), as presented in **Figure 2**. Mortality during hospitalization increased progressively with rising RAR values.

### 3.3 The association between RAR and mortality

Univariate logistic regression indicated that several variables were significantly associated with in-hospital mortality (all P < 0.05): age, gestational age, height, weight, STS-EACTS score, CPB time, AOX time, cyanotic CHD diagnosis, PLT, ALB,CREA, RDW-SD, and RAR. These results are detailed in **Table 2**.

**Table 2:** Factors associated with in-hospital mortality: univariate analysis and logistic regression.

Variables	Statistics	OR (95%CI)	P-value
Male			
No	1362 (37.48%)	1	
Yes	2272 (62.52%)	1.74 (0.98, 3.08)	0.0585
Age (day)	148.54 ± 96.05	0.99 (0.99, 0.99)	<0.0001
Gestational age(week)	38.56 ± 2.19	0.84 (0.78, 0.91)	<0.0001
Height	60.52 ± 13.00	0.91 (0.88, 0.93)	<0.0001
Weight	5.51 ± 2.57	0.58 (0.48, 0.69)	<0.0001
STS-EACTS score	2.58 ± 0.87	2.36 (1.69, 3.30)	<0.0001
CPB time	86.84 ± 58.27	1.01 (1.01, 1.01)	<0.0001
AOX time	46.54 ± 34.37	1.02 (1.01, 1.02)	<0.0001
CPB			
No	372 (10.24%)	1	
Yes	3262 (89.76%)	1.07 (0.46, 2.49)	0.8835
Cyanotic CHD diagnosis			
No	2607 (71.74%)	1	
Yes	1027 (28.26%)	4. 14 (2.47, 6.93)	<0.0001
WBC(x10^9/L)	10.04 ± 3.28	1.01 (0.94, 1.09)	0.746
RBC(x10^12/L)	4.26 ± 0.84	1.07 (0.80, 1.43)	0.6325
GLU(mmol/L)	5.21 ± 1.38	0.83 (0.66, 1.04)	0.1004
PLT(x10^9/L)	370.92 ± 119.60	0.99 (0.99, 1.00)	<0.0001
CREA(μmol/L)	26.74 ± 14.13	1.03 (1.02, 1.04)	<0.0001
RDW-SD(fL)	43.73 ± 8.29	1.08 (1.06, 1. 10)	<0.0001
ALB(g/L)	39.04 ± 4.55	0.81 (0.77, 0.85)	<0.0001
RAR(fL/(g/L))	1.15 ± 0.34	7.78 (4.84, 12.50)	<0.0001

OR, odds ratio; CI, confidence interval; RBC, red blood cells; WBC, white blood cells; PLT, platelets; GLU, glucose; RDW-SD, red cell distribution width standard deviation; ALB, albumin; CREA, Creatinine; RAR, red cell distribution width-to-albumin ratio; STS-EACTS, Society of Thoracic Surgeons-European Association for Cardio-Thoracic Surgery; CPB, cardiopulmonary bypass; AOX, aortic cross-clamping.

**Table 3** presents the results of the multivariate logistic regression analysis. When RAR was treated as a continuous variable, it remained significantly associated with increased mortality even after adjusting for a series of covariates. Based on the cut-off value, patients were stratified into two groups: those with RAR levels exceeding 1.35 were significantly associated with higher in-hospital mortality(P<0.0001). In Model 3, after adjusting for clinical confounders, patients with higher RAR (>1.35) had a significantly increased risk of in-hospital mortality compared to those with lower RAR (≤1.35) (OR 2.16, 95% CI 1.01-4.60, p = 0.0471).

**Table 3:** Associations between RAR and in hospital mortality.

Variables	Model 1		Model 2		Model 3	
	OR(95%CI)	P	OR(95%CI)	P	OR(95%CI)	P
RAR	7.78(4.84, 12.50)	<0.0001	5.01 (2.75, 9. 13)	<0.0001	2.37 (1.19, 4.74)	0.0145
RAR						
≤1.35	1		1		1	
>1.35	8.05 (4.66, 13.89)	<0.0001	4.47 (2.23, 8.96)	<0.0001	2.16 (1.01, 4.60)	0.0471

RAR, red cell distribution width-to-albumin ratio; CI, confidence interval; OR, odds ratio.

Model 1 adjust for: None.

Model 2 adjust for: male; age; gestational age; height; weight.

Model 3 adjust for: male; age; gestational age; height; weight; STS-EACTS score; AOX time; CPB; cyanotic CHD diagnosis; WBC; RBC; GLU; PLT.

These results demonstrate a significant and independent association between the red cell distribution width-to-albumin ratio (RAR) and mortality among infants who underwent cardiac surgery.

### 3.4 ROC analysis to evaluate the predictive performance of RAR for in-hospital mortality

**Figure 3:**
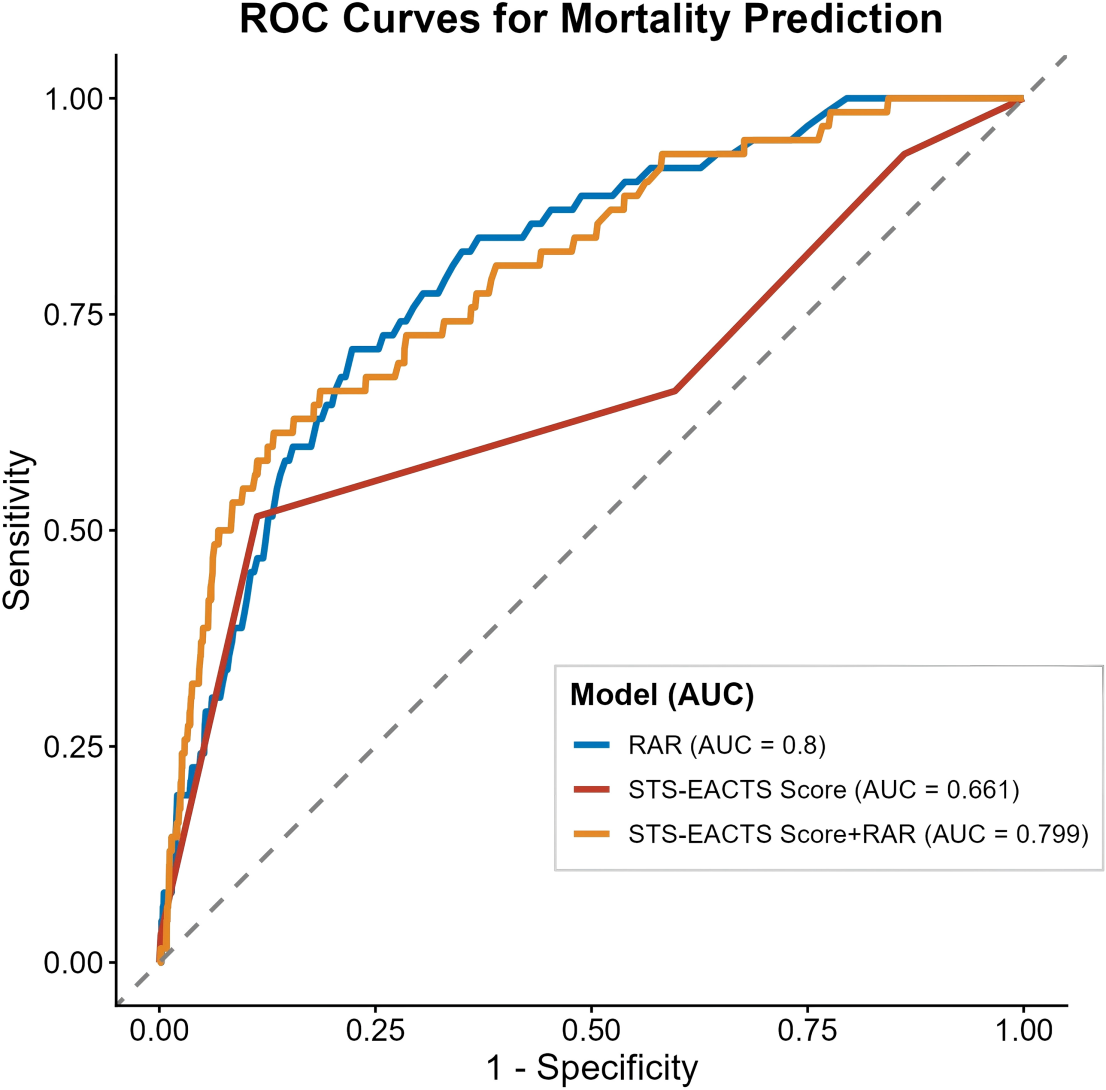
ROC curve-based assessment of the predictive value of STS-EACTS score, RAR, and Their Combination for in-hospital mortality in infants undergoing cardiac surgery.

A RAR cut-off point of 1.35 was established for identifying in-hospital mortality, with 70.97% sensitivity and 77.77% specificity. An AUC of 0.8 (95% CI: 0.748–0.853, p < 0.001) was obtained from the ROC analysis. These findings indicate that the RAR alone has predictive value for in-hospital mortality risk. We further calculated the incremental predictive value of RAR beyond the established STS-EACTS risk score: the AUC of the STS-EACTS score alone was 0.661, which increased to 0.799 after incorporating RAR into the model. The difference in AUC between the two models was statistically significant (DeLong test, P <0.001), confirming that RAR provides additional prognostic information beyond the conventional STS-EACTS risk score (**Table 4**, **Figure 3**).

**Table 4:** ROC analysis for the value of RAR in predicting in-hospital mortality.

Model	Cutoff	Specificity	Sensitivity	AUC (95% CI)
STS-EACTS Score	3.5	0.8870	0.5160	0.661(0.579-0.743)
RAR	1.345	0.7777	0.7097	0.8(0.748-0.853)
STS-EACTS Score+RAR	0.027	0.868	0.613	0.799(0.742-0.857)

CI, confidence interval; AUC, the area under the curve; STS-EACTS, Society of Thoracic Surgeons-European Association for Cardio-Thoracic Surgery; RAR, red cell distribution width-to-albumin ratio.

### 3.5 Subgroup analysis

**Figure 4:**
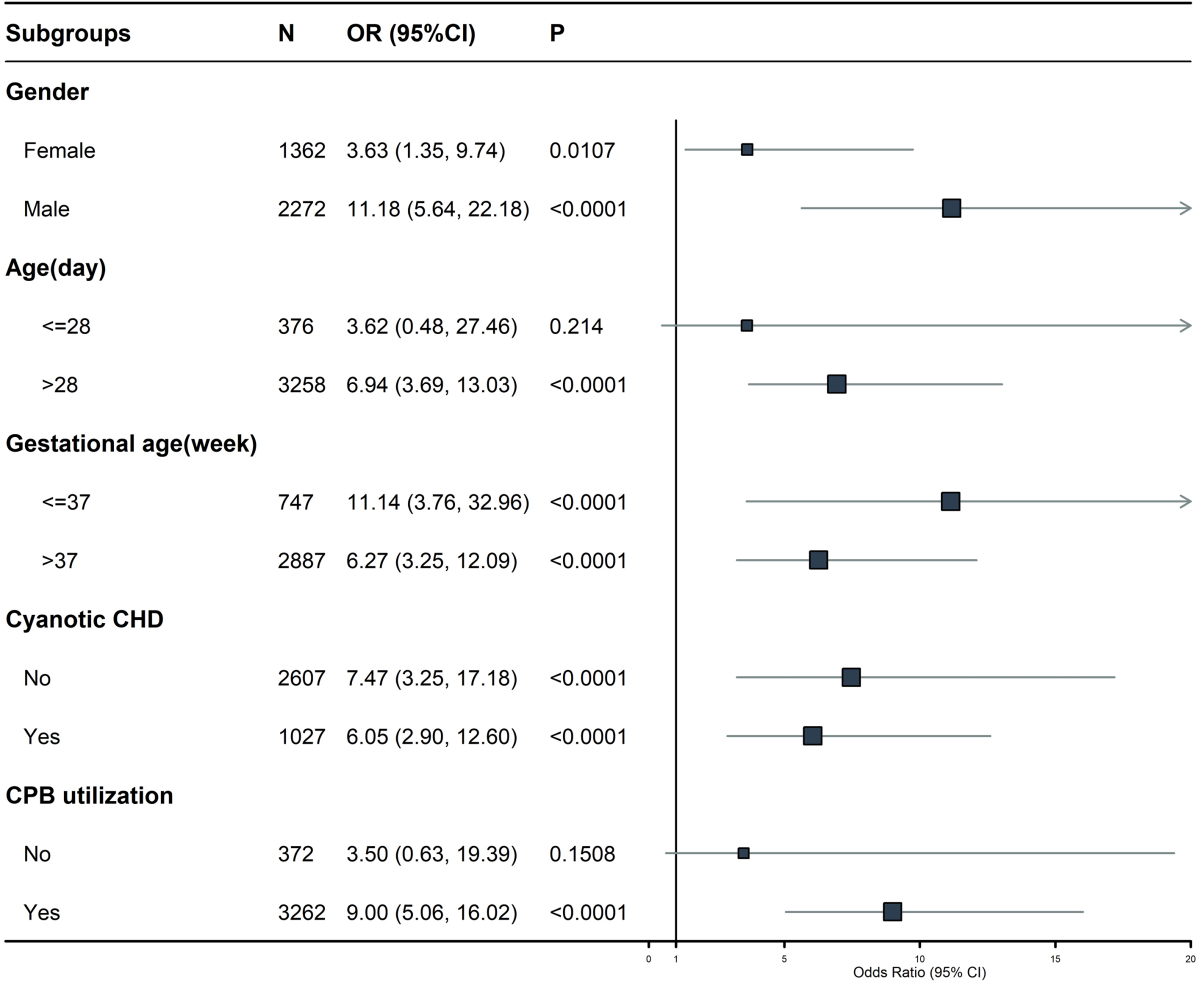
Forest plot of subgroup analyses for RAR and in-hospital mortality.

We performed subgroup analyses stratified by age, gender, gestational age, diagnosis of cyanotic CHD, and CPB utilization. Across all subgroups, the association maintained a consistent direction and indicated a positive correlation (OR > 1). However, no statistically significant effects were observed in the subgroups of age ≤ 28 days and CPB non-utilization. Subgroup analysis results are presented in **Figure 4** (forest plot).

## 4 Discussion

In a large cohort study, we examined the link between the novel combined biomarker RAR and mortality in infants undergoing cardiac surgery. Our results demonstrate that elevated RAR correlates with increased in-hospital mortality, whether assessed as a continuous or categorical variable. Even after adjustment for confounders, the elevated mortality risk associated with a higher RAR remained statistically significant. RCS analysis indicated a progressive increase in in-hospital mortality risk with higher RAR levels. Subgroup analyses for site-specific illnesses confirmed the robustness of these findings.

The exact biological mechanisms connecting increased RDW to mortality remain unclear. RDW acts as a general health indicator rather than a disease-specific marker, associating it with mortality across various diseases and the general population (13). Factors contributing to elevated RDW include inflammation (14), oxidative stress (15), telomere shortening (16), increased osmotic fragility of red blood cells (17), nutritional deficiencies (18), and erythropoietin deficiency or dysfunction. CPB duration, anatomical defect complexity, surgical repair intricacy, and cyanosis occurrence are all associated with the postoperative systemic inflammatory response (19). Inflammatory cytokines influence erythropoiesis by downregulating erythropoietin (Epo) gene expression in the kidneys and liver and hindering erythroid development in the bone marrow, leading to increased RDW (20). Oxidative stress also increases red blood cell fragility, slows erythroid maturation, and shortens red cell survival, representing another potential biological mechanism underlying elevated RDW (21). In the general population, an increased RDW has been identified as a robust and independent determinant of mortality risk. Beyond its prognostic significance, it also demonstrates a high negative predictive value for diagnosing various diseases (8).

In addition, elevations in RDW are commonly observed under several pathophysiological conditions. These include impairments in erythrocyte production, such as deficiencies in iron, vitamin B12, or folate, as well as disorders affecting hemoglobin. Heightened levels are also noted in clinical scenarios characterized by increased erythrocyte lysis or a history of blood transfusion. To counteract this elevation, rigorous surveillance and clinical management are essential to avert tissue hypoxia and hypoperfusion, while reducing unwarranted RBC transfusion throughout the perioperative phase (22). RDW is regularly assessed as a component of the commonly employed complete blood count and thus incurs no extra expense. Moreover, RDW is potentially modifiable (13). Elevated RDW levels in non-anemic patients warrant investigation into underlying causes, such as nutritional deficiencies or malabsorption (e.g., iron). Focused management is essential for specific risk factors that may worsen prognosis, including impaired renal function, physical inactivity, metabolic syndrome, and inflammation. When feasible, timely corrective measures should be implemented to restore RDW to within the normal reference range (23).

Albumin (ALB) is also identified as a marker associated with inflammation. Inflammatory processes enhance capillary permeability, causing serum albumin to escape, which results in the expansion of the interstitial space and an increased volume of albumin distribution. It decreases both the half-life and total mass of albumin. Together, these two factors contribute to hypoalbuminemia (24). Inflammation is associated with vascular disease and may cause vascular endothelial injury, with hypoalbuminemia representing an independent manifestation of the inflammatory process (25). Mortality risk increases progressively with declining albumin levels (26). Albumin has numerous important physiological functions and is essential for normal health (25).

Importantly, given that RAR is derived from the ratio of RDW to ALB, it may be modulated by numerous perioperative factors, thereby complicating its prognostic interpretation. First, systemic inflammation induced by CPB may increase RAR by elevating RDW through pro-inflammatory cytokine-mediated dysregulation of erythropoiesis (20, 28), while simultaneously reducing serum albumin through increased capillary leakage (24). Second, red blood cell transfusion increases RDW (29), whereas albumin administration increases serum albumin levels, both of which dynamically modify RAR values. Third, postoperative complications may further trigger systemic inflammation and organ dysfunction(hepatic and renal injury),leading to elevated RDW (20, 23) and reduced albumin (30, 31), thereby increasing RAR. Perioperative pathophysiological changes and clinical interventions interact in complex ways, and this interplay may jointly impact RAR levels as well as their association with in-hospital mortality.

Research indicates a positive correlation between the red cell distribution width to albumin ratio and overall mortality in critically ill patients (27). The present study substantiates earlier findings by indicating a connection between the RAR and mortality during hospitalization among infants who underwent cardiac surgery. Higher RAR values were observed to correlate with a greater probability of death.

Consequently, we proposed that integrating these two biomarkers might reflect inflammatory processes, nutritional deficits, and additional systemic disturbances, and may further be associated with mortality during the hospital admission period. The results of this study confirm the clinical utility of the RAR as a composite biomarker, which can be integrated into established clinical workflows. Moreover, they emphasize the potential benefits of using routinely available laboratory parameters in combination.

## 5 Conclusions

The present investigation was designed to examine the correlation between the RAR and mortality during hospital admission among infants who had received cardiac surgery. Additionally, an assessment of the predictive performance of this biomarker was conducted.

The findings indicated a correlation between increased RAR and elevated mortality rates, though certain limitations must be considered. First, being a retrospective analysis, this study is inevitably affected by selection bias, which may compromise the generalizability of our conclusions. Future studies should include external validation to confirm these findings. Second, the single baseline assessment of RAR limits its dynamic interpretation. Further investigation is warranted to determine whether continuous RAR measurement can accurately predict in-hospital mortality in this surgical population. Third, We were unable to obtain data on whether the infants had received relevant treatments at other hospitals shortly before admission. These factors may affect RDW and albumin levels, potentially confounding the RAR analysis, which represents a limitation of this study. Fourth, preoperative infection was indirectly assessed by preoperative white blood cell count without a standardized diagnostic definition, which may not fully reflect infectious status. Future studies with standardized infection criteria are warranted to more accurately evaluate the value of RAR.

## Data Availability

All relevant data are shown within the article, but original datasets cannot be shared because of involving infants’ privacy. Requests to access the datasets should be directed to Chongjian Zhang, zhangchongjian@gdph.org.cn.
