# Supplementary material for: RAR and infant mortality: a crucial link in cardiac surgery outcomes
Source: Front Immunol. 2026 Apr 16;17:1806409. doi: 10.3389/fimmu.2026.1806409 (PMC13128359; doi:10.3389/fimmu.2026.1806409)
Supplement: Supplementary file 1 [file Table1.docx]

| Supplementary Table 1 Description of missing variables | | | | | |
| --- | --- | --- | --- | --- | --- |
| **Variables** | | **Numbers** | | **Missing Rate(%)** | |
| Gestational age | | 1861 | | 26.06 | |
| GLU | | 81 | | 1.13 | |
| STS-EACTS Score | | 19 | | 0.27 | |
| CREA | | 13 | | 0.18 | |
| PLT | | 3 | | 0.04 | |
| Height | | 1 | | 0.01 | |
| Abbreviations GLU:glucose ;CREA:Creatinine;STS-EACTS:Society of Thoracic Surgeons-European Association for Cardio-Thoracic Surgery;PLT:platelets | | | | | |
| Supplementary Table 2 Variables Excluded Due to Collinearity (VIF) | | | | | |
| Variables | | Step 1 | | Step 2 | |
| RAR | | 1.7 | | 1.6 | |
| Male | | 1 | | 1 | |
| Age | | 2.3 | | 2.2 | |
| Gestational age | | 1 | | 1 | |
| Height | | 1.4 | | 1.4 | |
| Weight | | 1.6 | | 1.6 | |
| STS-EACTS score | | 1.2 | | 1.2 | |
| CPB time | | 5.2 | | NA | |
| AOX time | | 4.9 | | 1.5 | |
| CPB | | 1.5 | | 1.4 | |
| Cyanotic CHD diagnosis | | 1.4 | | 1.4 | |
| WBC | | 1.1 | | 1.1 | |
| RBC | | 1.6 | | 1.6 | |
| GLU | | 1 | | 1 | |
| PLT | | 1.1 | | 1.1 | |
| Abbreviations RBC:red blood cells ;WBC:white blood cells ;PLT:platelets ;GLU:glucose ;RAR:red cell distribution width-to-albumin ratio;STS-EACTS:Society of Thoracic Surgeons-European Association for Cardio-Thoracic Surgery;CPB:cardiopulmonary bypass;AOX:aortic cross-clamping. | | | | | |
